# A comparison of time-varying propensity score vs sequential stratification approaches to longitudinal matching with a time-varying treatment

**DOI:** 10.1186/s12874-024-02391-3

**Published:** 2024-11-13

**Authors:** Morgan Richey, Matthew L. Maciejewski, Lindsay Zepel, David Arterburn, Aniket Kawatkar, Caroline E. Sloan, Valerie A. Smith

**Affiliations:** 1 Department of Population Health Sciences, Duke University School of Medicine, Durham, 27705 NC USA; 2 Center of Innovation to Accelerate Discovery and Practice Transformation, Affairs Medical Center, Durham Veterans, Durham, NC USA; 3 Duke-Margolis Center for Health Policy, Duke University, Durham, NC USA; 4 Division of General Internal Medicine, Duke University, Durham, NC USA; 5 Department of Medicine, University of Washington, Seattle, WA USA; 6 Kaiser Permanente Washington Health Research Institute, Seattle, WA USA; 7 Department of Research and Evaluation, Kaiser Permanente Southern California, Pasadena, CA USA; 8 Department of Biostatistics and Bioinformatics, Duke University, Durham, NC USA

**Keywords:** Time varying propensity scores, Confounding, Bias, Matching, Precision, Sequential stratification

## Abstract

**Background:**

Methods for matching in longitudinal cohort studies, such as sequential stratification and time-varying propensity scores, facilitate causal inferences in the context of time-dependent treatments that are not randomized where patient eligibility or treatment status changes over time. The tradeoffs in available approaches have not been compared previously, so we compare two methods using simulations based on a retrospective cohort of patients eligible for weight loss surgery, some of whom received it.

**Methods:**

This study compares matching completeness, bias, coverage, and precision among three approaches to longitudinal matching: (1) time-varying propensity scores (tvPS), (2) sequential stratification that matches exactly on all covariates used in tvPS (SS-Full) and (3) sequential stratification that exact matches on a subset of covariates (SS-Selected). These comparisons are made in the context of a deep sampling frame (50:1) and a shallow sampling frame (5:1) of eligible comparators. A simulation study was employed to estimate the relative performance of these approaches.

**Results:**

In 1,000 simulations each, tvPS retained more than 99.9% of treated patients in both the deep and shallow sampling frames, while a smaller proportion of treated patients were retained for SS-Full (91.6%) and SS-Selected (98.2%) in the deep sampling frame. In the shallow sampling frame, sequential stratification retained many fewer treated patients (73.9% SS-Full, 92.0% SS-Selected) than tvPS yet coverage, precision and bias were comparable for tvPS, SS-Full and SS-Selected in the deep and shallow sampling frames.

**Conclusion:**

Time-varying propensity scores have comparable performance to sequential stratification in terms of coverage, bias, and precision, with superior match completeness. While performance was generally comparable across methods, greater match completeness makes tvPS an attractive option for longitudinal matching studies where external validity is highly valued.

## Background

Cohort studies are powerful tools for generating real-world evidence on long-term outcomes when randomization is infeasible or unethical or when trial follow-up is limited in duration. There are various approaches to reduce confounding by matching treated patients to comparators, each with tradeoffs in bias, coverage, and generalizability. Most evaluations of matching methods have focused on cross-sectional studies where crossover is rare or impossible. With greater use of longitudinal cohort designs in which eligibility for treatments and treatment status are time-dependent, there is increased interest in the use of matching methods for these contexts but few comparisons have been undertaken to date [1]. Matching patients with appropriate comparators in a longitudinal context can be challenging depending on the similarity of untreated patients to the treated patients, the size of the untreated sampling frame, and the frequency and measurement completeness of covariates.

Simulation studies using cross-sectional data have estimated the tradeoffs in sample loss, bias, and precision when varying the numbers of comparators allowed to match to each treated patient, matching order, the use of calipers of different widths, score trimming, and matching with or without replacement [2–5]. Selection of variables to include in a propensity score specification has been extensively studied, with consensus that only confounders and predictors of the outcome should be included [6], though approaches vary [7–11]. Prior work has also found that bounding matches with calipers results in better covariate balance than unbounded matching. (11–12) A caliper that is 0.2 of the standard deviation of the logit of the propensity scores often represents a reasonable bound [3]. Matching with replacement may reduce bias when comparators are sparse (in terms of number available or comparability) [13], but can increase bias when comparable comparators are plentiful [12]. Matching with replacement also requires advanced variance estimators to obtain appropriate confidence intervals [14]. 

A less explored constraint is sample size, as well as the size of the untreated comparator in relation to the treatment group. The relative sample frame prior to matching may be relatively shallow (1–5 untreated per treated patient) if the treatment is common or eligibility criteria are strict. Alternatively, the size of the untreated sampling frame may be relatively deep (50–500:1) if the treatment is rarely offered or rarely accepted, despite eligibility criteria being inclusive. In this report, we compare performance with shallow and deep sampling frames of potential comparators to evaluate the tradeoffs in internal and external validity.

The proposed work builds upon prior research by comparing matching methods in the context of time-varying treatment eligibility and time-varying confounding and is partly motivated by prior experience with cohort matching for bariatric surgery that is rarely offered (in percentage terms) to the many eligible patients [15]. We extend a hazard-based, time-varying propensity score (tvPS) approach proposed by Lu in 2005^16^ to allow for replacement of comparators and compare this method to sequential stratification (SS) proposed by Schaubel et al. in 2006 [17] in a simulation study. Propensity score methods match patients on a score derived from combining observable confounders and risk factors of the outcome, while SS matches patients directly on covariates. Our simulation is motivated by our comparative effectiveness studies of the impact of bariatric surgery on weight loss, healthcare expenditures, and survival [18–25]. 

We use this simulation to compare matching completeness (percent of bariatric surgery patients with a match), precision, bias, and coverage of treatment effect estimates and their resulting confidence intervals. We present these comparisons across one tvPS and two SS methods in shallow (1:5) and deep (1:50) sampling frames of non-surgical comparators. The two SS alternatives represent an application with a full specification of covariates identical to that used in the PS models (SS-Full), and a more pragmatic approach that uses a restricted set of covariates selected to minimize sample loss (SS-Selected) that can occur in exact matching contexts due to the curse of dimensionality [26]. 

In section 2, we describe our motivating example, review propensity scores and sequential stratification approaches in section 3, describe our simulation and results in section 4, and provide concluding remarks in section 5 and point to areas for future research.

## Methods

### Motivating example

Our motivating example is based on a longitudinal cohort study of bariatric surgery in eligible patients enrolled in Kaiser Permanente-Washington state (KPWA), which is longitudinal in two respects. First, retrospectively identified participants were eligible for and/or received surgery over a 7-year period. Second, outcomes were compared for several years following inclusion. Between 2012 and 2019, 1,570 KPWA enrollees received bariatric surgery. In the motivating study, a sampling frame of comparators was constructed, comprising 103,615 enrollees who were guideline-eligible for bariatric surgery, e.g., BMI > 35. Both cases and comparators were required to have at least 1 year of enrollment prior to their entry into the cohort.

We chose total weight loss percent (TWL%) as our outcome, classified variables as confounders, predictors of the outcome, and predictors of the exposure through consultation with subject matter experts. Covariates considered confounders or predictors of the outcome were eligible for use in for matching, covariates considered to be primarily predictors of the exposure only were not used for matching. As a result, we selected 14 covariates for potential inclusion in matching, including demographic factors such as age and sex, as well as comorbidities including diabetes, insulin use and diagnosed depression. To estimate 2-year TWL% to inform our simulations, we restricted the cohort to those with weight values measured at 2 years (*N* = 717 surgical patients). The characteristics of the restricted KPWA population are detailed in Table 1, with the variables used for the different matching methods in Table 2.

**Table 1:** Characteristics of the KPWA study population

	Cases (*N* = 717)	Comparators (*N* = 42,818)
Covariate	N (%)	N (%)
Diabetes	301 (42.0%)	9,720 (22.7%)
Female	582 (81.2%)	27,660 (64.6%)
Insulin use	147 (20.5%)	3,939 (9.2%)
Atrial fibrillation	34 (4.7%)	2,141 (5.0%)
Cirrhosis	5 (0.7%)	257 (0.6%)
Depression	392 (54.7%)	12,289 (28.7%)
Dyslipidemia	394 (55.0%)	10,319 (24.1%)
Eating disorder	23 (3.2%)	214 (0.5%)
Fatty liver disease	70 (9.8)	1,158 (2.7%)
Gastro-esophageal reflux disease	390 (54.4)	6,380 (14.9%)
Hiatal hernia	164 (22.9%)	471 (1.1%)
Myocardial infarction	50 (7.0%)	1,798 (4.2%)
History of stroke, TIA, cerebrovascular disease	17 (2.4%)	1,113 (2.6%)
Body Mass Index, mean (SD)	44.8 (6.57)	39.8 (5.45)
Quan/Gagne combined comorbidity score, mean (SD)	0.4 (1.32)	0.9 (1.99)
Risk of pulmonary embolism	56 (7.8)	2,312 (5.4%)
Age (years), mean (SD)	52.9 (11.58)	51.9 (14.80)

**Table 2:** Variable inclusion by matching Method compared

Category	Variable	SS-Selected	SS-All	tvPS
Risk Stratification	Quan/Gagne score*	X	X	X
Anthropometric	BMI Category	X	X	X
	Age	X	X	X
	Sex	X	X	X
Diabetes	Diabetes Status	X	X	X
	Insulin Use	X	X	X
Hepatic / Cardiovascular	Myocardial Infarction		X	X
	Fatty Liver Disease		X	X
	History of VTE		X	X
	Cirrhosis		X	X
	Stroke, TIA, or CVA**		X	X
	Atrial Fibrillation		X	X
Digestive	Eating Disorder		X	X
Mental Health	Diagnosed Depression		X	X

*Quan/Gagne = Risk scoring algorithm based on Gagne score using ICD10 diagnosis codes in administrative data [27].

**Stroke, TIA, CVA are defined as a history of stroke and/or: transient ischemic attack, cerebrovascular accident.

### Overview of time-varying propensity scores and sequential stratification

Briefly, we describe two approaches to longitudinal matching in the setting of cohort studies, our implementation of these approaches, and the performance measures we will use to compare them.

#### Time-varying propensity scores

Time-varying propensity score approaches balance the marginal covariate distribution between treated patients and comparators at each time point during the study using a propensity score derived from a Cox proportional hazard model [15]. The hazard of receiving a treatment is estimated in a population of patients receiving the treatment and eligible comparators who have not yet (but may later have) received the treatment, based on observed confounders and predictors of the outcome at each time point [6]. Based on these estimates of the hazard of treatment, time-specific propensity scores are calculated for each treated patient and comparators. As is typical with matching-based approaches, this method assumes no unobserved confounding. While the original approach by Lu did not include matching with replacement, we evaluate the performance of this method when replacement is allowed. We used a standardized approach (SAS PROC PSMATCH) that selects the comparator with the closest propensity score to a treated patient regardless of whether that comparator has been matched to a different patient at an earlier time point. The equation for the time varying propensity score, estimated as the hazard of receiving treatment, can be written as $\:\:{\lambda\:}_{i}^{T}\left(s\right|\varvec{\gamma\:})={\lambda\:}_{0}^{T}(s){e}^{\left\{\varvec{\gamma\:}{\prime\:}{\varvec{Z}}_{i}\left(s\right)\right\}}$ [1]. 

As described by Lu [15], the hazard of treatment for patient *i* at time point *s*, who has covariate profile $\:{\varvec{Z}}_{i}\left(s\right)$ at time *s* is estimated by $\:\:{\lambda\:}_{i}^{T}\left(s|\varvec{\gamma\:}\right),\:$where $\:\varvec{\gamma\:}$represents the vector of parameters to be estimated. The values in $\:{\varvec{Z}}_{i}\left(s\right)$ are allowed to vary at each recorded encounter and are assumed to remain constant between encounters. Comparators are defined as participants who are still eligible for treatment (e.g., bariatric surgery) at time point *s* but have yet to receive it. To translate the hazards to the more familiar propensity score framework bounded between zero and one, the highest estimated hazard of receiving treatment was used as the divisor for all other scores, yielding a bounded score to use in matching.

For our implementation of this approach, comparators were required to have an eligible encounter within the 6-month period prior to a case’s surgery date to be eligible for matching. Calipers were employed to limit the difference in propensity score between cases and a potential matched comparator to 0.2 of the standard deviation of the logit of the transformed propensity score within the common support area. A greedy nearest-neighbor approach with randomly sorted data was used to match. Individuals who had surgery during the study period were allowed to act as comparators prior to their surgery date.

#### Sequential stratification

Sequential stratification [16] mimics a randomized trial in which eligible patients are randomly assigned to treatment over many years, as would be seen during a long enrollment period of a trial. Each patient choosing treatment is linked to a risk set of eligible comparators who have similar characteristics observed close in time to treatment initiation. Chosen categorical variables are matched exactly, while continuous variables can be coarsened via categories or ‘bands’. Within each risk set, the most similar matches can be identified on the basis of minimizing a Mahalanobis distance function for continuous covariates. (28–29)

We present two different covariate specifications for sequential stratification. First, a full specification of covariates (SS-Full) was applied, where covariates matched on were identical to those used in the propensity score specification. Second, a restricted covariate specification was used (SS-Selected), based on clinical knowledge of the most important confounders of the relationship between bariatric surgery and weight loss to minimize the percentage of treated patients who do not have a match (i.e., maximize match completeness). This reflects a common challenge, often referred to as the curse of dimensionality, when exact matching in high-dimensional datasets: the number of variables that can be used may have to be constrained to retain a high proportion of matches [24]. This specification necessitates a ranking of variables for SS-Selected by the study team, often by the hypothesized strength of confounding potential, based on investigator judgment, and is a limitation of exact matching methods in high-dimensional settings.

### Simulation studies

#### Data generating process

We generated simulated datasets using the mean vector and correlation matrix of a rich, real-world database containing a very deep sampling frame (average of 60 comparators per treated patient) of bariatric surgery recipients and patients eligible for bariatric surgery in Kaiser Permanente Washington (KPWA). Basing our simulation on the real-world correlation among covariates allows us to represent the complex interplay the potential confounding factors we wish to control for may have among each other. This approach is intended to challenge these methods by testing their ability to support causal inference in the highly variable, real-world data used by investigators seeking to apply advanced matching methodologies.

The relative effects on matching completeness, bias, coverage, and precision by competing matching approaches were estimated by varying the inputs to the simulation, including associations between exposure, outcome, and covariates. We utilize the baseline correlation structure among all observed variables present in the KPWA sampling frame of bariatric surgery cases and non-surgical comparators separately and simulate new populations that share their baseline characteristics (using SAS PROC SIMNORMAL). The values of continuous covariates were assigned by the output of PROC SIMNORMAL, while categorical covariates were coded for cases and controls separately; if the predicted probability from PROC SIMNORMAL exceeded the prevalence in the KPWA data, the variables were assigned a positive value. Sixteen encounters were simulated for comparators (a group that includes some future cases acting as comparators prior to their surgery date) to mimic the multiple eligible time points observed in longitudinal cohort studies. At each encounter, time-invariant covariates (such as sex) remained stable while other covariates (such as BMI) were allowed to vary.

To inform simulation values, two-year TWL% outcomes were assigned using results from a linear regression model in the KPWA data that estimated total percentage weight loss as a function of observed covariates, including whether the individual received bariatric surgery. The parameter estimates from this model were used to assign percent total weight loss outcomes in the simulated dataset, with the estimated treatment effect from this regression used as the “true value” of the treatment effect in the simulation approaches. Random variation was incorporated by generating the simulated TWL% outcome as a normally distributed variable with mean based on a simulated individual’s covariate profile and standard deviation of 0.34%.

We perform analyses in two separate simulated cohorts. In the first cohort, we use a deep sampling frame of comparators, randomly simulating 50 times as many non-surgical comparators as bariatric surgery patients. In the shallow sampling frame, we simulate the same number of cases but with only 5 times as many randomly simulated comparators.

We simulated total populations of 1,570 cases and their comparators based on the sampling frame (7,850 comparators for shallow, 78,500 for deep), with 1,000 unique populations for each sampling pool. In each individual simulation, a unique set of cases and comparators were randomly generated with covariates mimicking the correlation structure of the KPWA cohort, a propensity score estimated and assigned to cases and comparators at each eligible time point, and tvPS and SS matching approaches employed to match cases to an eligible comparator, with replacement, if available. Once all cases who could be matched to a comparator had been matched by each method in each sampling pool, a linear regression model (using normal distribution and identity link in PROC GENMOD) was employed within each simulated dataset to estimate the association between bariatric surgery and 2-year TWL% comparing cases to matched comparators, adjusted for the full set of covariates used by tvPS and SS-Full. (Table 2). To account for the correlation structure induced by replacement of comparators in each approach in our treatment effect estimate, we utilized empirical sandwich standard errors clustered on unique individuals. We included full covariate adjustment to understand if regression adjustment can overcome the limitations of SS-Selected, which omitted known confounders from the matching process.

### Performance of methods

We compared the matching performance of the two methods in four ways: matching completeness (number and percent of surgical cases matched to a non-surgical comparators), coverage of 95% confidence intervals, precision (using Confidence-Interval Ratios [CIR], calculated as upper-CI divided by lower-CI) and bias of the estimated treatment effect of bariatric surgery TWL%. Coverage was evaluated by reporting the number and percent of simulations that contain the true value in their 95% confidence interval. Precision, when the confidence interval included the true value, was evaluated using CIR, defined as the ratio of the upper 95% confidence interval to the lower 95% confidence intervals of the regression model estimate of the treatment effect of bariatric surgery on TWL%. Bias in the estimated effect was assessed by the distance and direction between the effect estimate and the true value among all simulations.

## Results

### Overall results summary

All matching methods achieved excellent coverage, low bias, and good precision in both sampling pools, with tvPS achieving the highest proportion of matched cases, with comparable coverage, bias, and precision (Table 3).

**Table 3:** Relative performance of matching methods: number/proportion of matches, coverage, precision, bias

50:1 (Deep Sampling Frame)						5:1 (Shallow Sampling Frame)				
	**Cases matched** **(mean)**	**Percent matched** **(mean)**	**Coverage**	Precision* **(mean)**	**Bias (mean)**	**Cases matched (mean)**	**Percent matched (mean)**	**Coverage**	**Precision** ^*^ **(mean)**	**Bias (mean)**
tvPS	1,570	100.0%	94.7%	1.05	0.004%	1,570	100.0%	95.1%	1.05	0.02%
SS - Selected	1,541	98.2%	95.3%	1.06	0.01%	1,445	92.0%	94.8%	1.06	0.003%
SS - Full	1,438	91.6%	96.1%	1.05	0. 01%	1,160	73.9%	94.1%	1.06	-0.01%

^*^Precision expressed as confidence interval ratio, calculated as the ratio of the upper confidence interval to the lower confidence interval

### Simulation in Deep (50:1) sampling frame of untreated patients

Across the 1,000 simulated datasets in the deep sampling frame of 50 non-surgical comparators for every surgical patient, time-varying propensity scores with replacement (tvPS) matched 100% of cases, while SS-Selected matched 98.2% of cases and SS-Full matched the fewest (91.6%) of cases, as expected (Table 3).

**Fig. 1:**
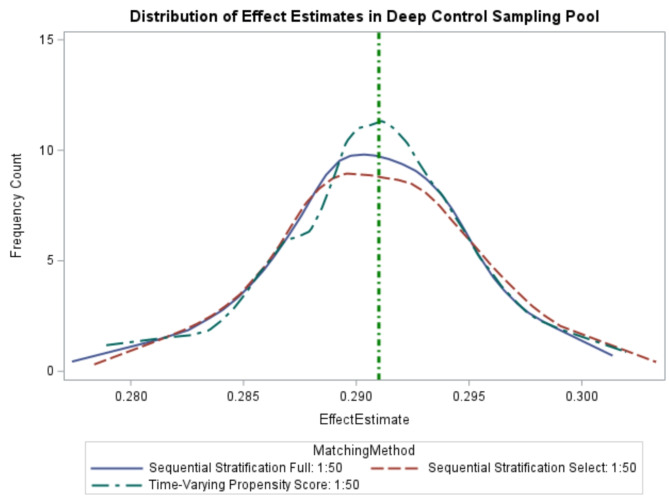
Distribution of Effect Estimates in Deep Comparators (50:1) Sampling Pool

The true value of 2-year TWL% was 29.1%. Across the 1,000 simulations, matching with tvPS resulted in 947 (94.7%) simulations of the TWL% association with regression-estimated 95% confidence intervals containing the true value. The tvPS approach resulted in a mean estimated effect of 29.1%, featuring good precision (Confidence Interval Ratio [CIR]: 1.05), and minimal bias (0.004%). Across these same simulations, SS-Selected matching method resulted in 953 (95.3%) confidence intervals that contained the true value, with a mean estimated effect of 29.1%, with similar precision (CIR: 1.06) and bias (0.01%). Using SS-Full, 961 (96.1%) of confidence intervals contained the true value, with a mean regression estimate of 29.1%, with similar precision (CIR: 1.05) and bias (0.01%). The distribution of effect estimates was similar between the three approaches, with a slightly tighter distribution observed for tvPS compared to SS-Full and SS-Selected (Fig. 1).

### Simulations in the shallow (5:1) sampling frame of untreated patients

In the 1,000 simulated datasets in the shallow sampling frame, time-varying propensity scores with replacement (tvPS) again matched greater than 99.9% of cases (Table 3), while SS-Selected matched 92.0% of cases, and SS-Full matched the fewest (73.9%) of cases, as expected. (Table 3)

**Fig. 2:**
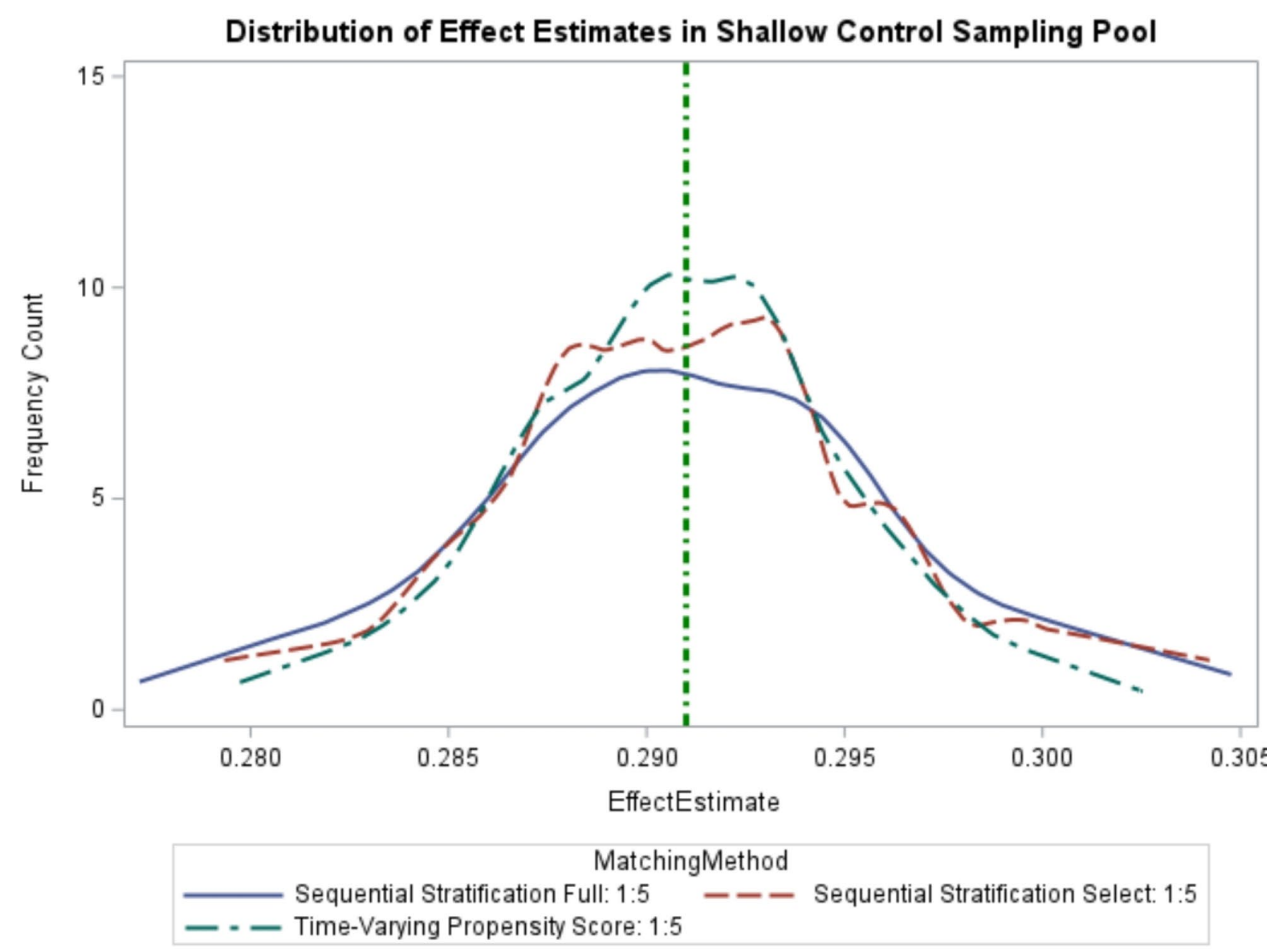
Distribution of Effect Estimates in Shallow Comparators (5:1) Sampling Pool

Across the 1,000 simulations, using tvPS resulted in 951 (95.1%) confidence intervals containing the true value, with a mean estimate of 29.1%, featuring good precision (CIR: 1.05) and a bias estimate of 0.02%. SS-Selected approaches resulted in 948 (94.8%) confidence intervals containing the true value, with a mean estimate of 29.1%, with similar precision (CIR: 1.06) and a bias estimate of 0.003%. Using SS-Full, 941 (94.1%) confidence intervals contained the true value, with a mean regression estimate of 29.1%, with similar precision (CIR: 1.06) and bias (-0.01%). The patterns of the distribution of effect estimates were similar in the shallow sampling pool, with the tightest distribution observed with tvPS followed by SS-Selected. (Fig. 2).

## Discussion

There are many considerations when matching treated patients to comparators in longitudinal cohort studies, as there are several alternative methods and the tradeoffs inherent in each method are not yet well understood. In this report, we compare the performance of two matching methods: (1) sequential stratification that exact-matches on categorical covariates and uses Mahalanobis distances to select comparators with the closest value for continuous covariates, and (2) time-varying propensity score with replacement. Performance was compared in terms of matching completeness, confidence interval coverage, bias, and precision, when there were many (50:1) or few (5:1) potential comparators per treated patient. Time-varying propensity scores adjust for time-varying confounding, account for selection bias, and share with sequential stratification the assumption that all pertinent covariates are observed and correctly specified.

In the deep sampling frame of comparators, our simulation results found that tvPS is superior in terms of matching completeness for treated patients (100% compared to 92–98% in SS) compared to sequential stratification and comparable in terms of precision, coverage and bias compared to sequential stratification. Matching completeness (and the expectation of generalizability due to greater retention of the treated cohort) was better than the two sequential stratification approaches because tvPS matches based on a composite score rather than exact matching on specific covariates. Matching completeness was poorest with sequential stratification that incorporated the full suite of covariates (SS-Full) due to the curse of dimensionality, with a loss of 8% of treated patients compared to 2% loss with SS-Selected, which used a subset of covariates to limit sample loss. All approaches had near 95% coverage in the deep sampling frame.

In the shallow (5:1) sampling frame of comparators, the potential tradeoff in internal and external validity (aka generalizability) was more acute, especially for SS-Full where loss of the treatment group increased from 8 to 26%. The sample loss in the shallow sampling frame for SS-Selected increased from 2 to 8%, with precision and bias estimates similar to SS-Full and tvPS. Time-varying PS again matched nearly 100% of treated patients, with good coverage, precision, and a low bias estimate. While performance was generally comparable across methods in this simulation, greater match completeness, with the potential for improved generalizability, makes tvPS an attractive option for longitudinal matching studies. Sequential stratification that matches on the complete set of covariates for tvPS sacrificed generalizability to retain strong internal validity, so may not be optimal for contexts when the treatment is common (e.g., statins) or eligibility criteria are strict (e.g., anti-obesity medications).

Our work has several strengths, including basing our simulation on a large, real-world database of bariatric surgery patients, and those contemporaneous patients eligible for bariatric surgery based on measured BMI. We estimated the correlation matrix between a large number of covariates to reflect real-world complexities of covariate relationships. Most importantly, we compared two approaches to longitudinal matching in deep and shallow sampling frames of comparators to illustrate how results vary by depth of the sampling frame.

There are several limitations to our work, including the assumption of a constant treatment effect across the population. This assumption may result in an underestimate of the bias in the average effect estimate for methods that match less than 100% of patients. In the scenario of a treatment effect that is different among patients who are matched compared to those who are dropped due to not being matched, bias of the average effect estimate can be expected to increase, and results may not generalize well to the full population. Our definition of the true TWL% relies on a regression model approach that includes functional form decisions identical to our final regression model – the implication being that incorrectly specified regression models may perform differently. We made this simplification to understand relative performance of each method under controlled conditions. While we included 14 covariates, in two different sampling pools (50:1, 5:1, comparators to controls) to illustrate the performance of these methods, a larger suite of covariates or different ratios of comparators to controls could influence relative performance. Furthermore, investigators wishing to use binary or ordinal outcome measures will not be able to utilize the regression adjustment approach in our final model, as the expectation of collapsibility is not present when using a logit link function. The prevalence of covariates in the case and comparator groups also requires careful attention when building the hazard model that generates the final propensity score, if there are conditions common in one group but rare in another, models can fail to converge or assign extreme propensity values for some combinations of covariates. We recommend inclusion and exclusion criteria be carefully applied to ensure appropriately comparable groups prior to initiation of the matching process. Lastly, we did not simulate unmeasured confounders. We hypothesize that uncontrolled confounding may alter the performance of these models, but it is unclear whether the effect of uncontrolled confounding on model performance differs by matching approach. Future investigation of these methods using simulation approaches that can quantify the consequences of including unmeasured confounders with varying correlations with observed variables, the impact of important confounders that are exceedingly rare in one group compared to the other, as well as the effect of comparator replacement strategies would further inform the comparative utility of these two methods.

As interest grows in causal inference approaches in the context of time-dependent treatment where patient eligibility or treatment status changes over time, rigorous interrogation of the tradeoffs of new and existing methods are important avenues of inquiry. These results suggest that time-varying propensity scores have comparable bias and precision to sequential stratification when comparators are plentiful and have greater match completeness, thus optimizing external validity and generalizability with little loss of internal validity. When there are sufficient comparators available, sequential stratification may better preserve covariate balance in subgroups than tvPS when conducting heterogeneity of treatment effect analysis, if subgroups are defined on covariates that were part of the sequential stratification exact matching specification, which merits evaluation in future work. When evaluating the average treatment effect in contexts when comparators are scarce, time-varying propensity scores perform well with minimal bias tradeoff, making them an appealing alternative in many studies needing to utilize matching in a longitudinal context with time-varying eligibility and confounding covariate patterns.

## Conclusions

In our simulations, sequential stratification and time-varying propensity score approaches provided comparable results. Time-varying propensity scores likely enable a larger number of covariates be controlled on average without loss of sample, while sequential stratification may provide tighter balance on a specific, more limited set of covariates, ideal for potential subgroup analyses. We recommend consideration of the study-specific needs, sample sizes, and data availability when determining which to use.

## Data Availability

The datasets generated and analyzed during this study are not publicly available. Data were received from Kaiser Permanente Washington under a data use agreement that does not allow subsequent disclosure. SAS code for implementing tvPS is available on GitHub at https://github.com/DocRichey/tvPS/blob/main/README.md.

## References

[1] Thomas LE, Yang S, Wojdyla D, Schaubel DE. Matching with time-dependent treatments: a review and look forward. Stat Med. 2020;39(17):2350–70. 10.1002/sim.8533. 32242973 10.1002/sim.8533 PMC7384144

[2] Austin PC. Statistical criteria for selecting the optimal number of untreated subjects matched to each treated subject when using many-to-one matching on the Propensity score. Am J Epidemiol. 2010;172(9):1092–7. 10.1093/aje/kwq224. 20802241 10.1093/aje/kwq224 PMC2962254

[3] Austin PC. Optimal caliper widths for propensity-score matching when estimating differences in means and differences in proportions in observational studies. Pharm Stat. 2011;10(2):150–61. 10.1002/pst.433. 20925139 10.1002/pst.433 PMC3120982

[4] Stürmer T, Rothman KJ, Avorn J, Glynn RJ. Treatment effects in the Presence of Unmeasured Confounding: dealing with observations in the tails of the propensity score Distribution—A Simulation Study. Am J Epidemiol. 2010;172(7):843–54. 10.1093/aje/kwq198. 20716704 10.1093/aje/kwq198 PMC3025652

[5] Austin PC. The performance of different propensity score methods for estimating marginal hazard ratios. Stat Med. 2013;32(16):2837–49. 10.1002/sim.5705. 23239115 10.1002/sim.5705 PMC3747460

[6] Brookhart MA, Schneeweiss S, Rothman KJ, Glynn RJ, Avorn J, Stürmer T. Variable selection for propensity score models. Am J Epidemiol. 2006;163(12):1149–56. 10.1093/aje/kwj149. 16624967 10.1093/aje/kwj149 PMC1513192

[7] Baser O. Too much ado about Propensity score models? Comparing methods of propensity score matching. Value Health. 2006;9(6):377–85. 10.1111/j.1524-4733.2006.00130.x. 17076868 10.1111/j.1524-4733.2006.00130.x

[8] Steiner PM, Cook D. Matching and propensity scores. The Oxford Handbook of Quantitative Methods (vol 1): foundations. Oxford library of psychology. Oxford University Press;; 2013. pp. 237–59.

[9] VanderWeele TJ. Principles of confounder selection. Eur J Epidemiol. 2019;34(3):211–9. 10.1007/s10654-019-00494-6. 30840181 10.1007/s10654-019-00494-6 PMC6447501

[10] VanderWeele TJ, Shpitser I. On the definition of a confounder. Ann Stat. 2013;41(1):196–220. 25544784 10.1214/12-aos1058 PMC4276366

[11] Austin PC. An introduction to Propensity score methods for reducing the effects of confounding in Observational studies. Multivar Behav Res. 2011;46(3):399–424. 10.1080/00273171.2011.568786. 10.1080/00273171.2011.568786 PMC3144483 21818162

[12] Austin PC. A comparison of 12 algorithms for matching on the propensity score. Stat Med. 2014;33(6):1057–69. 10.1002/sim.6004. 24123228 10.1002/sim.6004 PMC4285163

[13] Dehejia RH, Wahba S. Propensity score-matching methods for nonexperimental causal studies. Rev Econ Stat 11.

[14] Austin PC, Cafri G. Variance estimation when using propensity-score matching with replacement with survival or time-to-event outcomes. Stat Med. 2020;39(11):1623–40. 10.1002/sim.8502. 32109319 10.1002/sim.8502 PMC7217182

[15] Campos GM, Khoraki J, Browning MG, Pessoa BM, Mazzini GS, Wolfe L. Changes in utilization of bariatric surgery in the United States from 1993 to 2016. Ann Surg. 2020;271(2):201–9. 10.1097/SLA.0000000000003554. 31425292 10.1097/SLA.0000000000003554

[16] Lu B. Propensity score matching with time-dependent covariates. Biometrics. 2005;61(3):721–8. 10.1111/j.1541-0420.2005.00356.x. 16135023 10.1111/j.1541-0420.2005.00356.x

[17] Schaubel DE, Wolfe RA, Port FK. A sequential stratification method for estimating the Effect of a time-dependent experimental treatment in Observational studies. Biometrics. 2006;62(3):910–7. 10.1111/j.1541-0420.2006.00527.x. 16984335 10.1111/j.1541-0420.2006.00527.x

[18] Smith VA, Arterburn DE, Berkowitz TSZ, et al. Association between Bariatric Surgery and long-term Health Care expenditures among veterans with severe obesity. JAMA Surg. 2019;154(12):e193732–193732. 10.1001/jamasurg.2019.3732. 31664427 10.1001/jamasurg.2019.3732 PMC6822094

[19] Maciejewski ML, Livingston EH, Smith VA, et al. Survival among high-risk patients after bariatric surgery. JAMA. 2011;305(23):2419–26. 10.1001/jama.2011.817. 21666276 10.1001/jama.2011.817

[20] Arterburn DE, Olsen MK, Smith VA, et al. Association between bariatric surgery and long-term survival. JAMA. 2015;313(1):62–70. 10.1001/jama.2014.16968. 25562267 10.1001/jama.2014.16968

[21] Maciejewski ML, Arterburn DE, Van Scoyoc L, et al. Bariatric surgery and long-term durability of weight loss. JAMA Surg. 2016;151(11):1046–55. 10.1001/jamasurg.2016.2317. 27579793 10.1001/jamasurg.2016.2317 PMC5112115

[22] Sudan R, Maciejewski ML, Wilk AR, Nguyen NT, Ponce J, Morton JM. Comparative effectiveness of primary bariatric operations in the United States. Surg Obes Relat Dis. 2017;13(5):826–34. 10.1016/j.soard.2017.01.021. 28236529 10.1016/j.soard.2017.01.021

[23] Maciejewski ML, Smith VA, Berkowitz TSZ, et al. Association of Bariatric Surgical procedures with changes in unhealthy Alcohol Use among US veterans. JAMA Netw Open. 2020;3(12):e2028117. 10.1001/jamanetworkopen.2020.28117. 33346846 10.1001/jamanetworkopen.2020.28117 PMC7753905

[24] Maciejewski ML, Smith VA, Berkowitz TSZ, et al. Long-term opioid use after bariatric surgery. Surg Obes Relat Dis. 2020;16(8):1100–10. 10.1016/j.soard.2020.04.037. 32507657 10.1016/j.soard.2020.04.037 PMC7423624

[25] Maciejewski ML, Arterburn DE, Berkowitz TSZ, et al. Geographic Variation in Obesity, behavioral treatment, and bariatric surgery for veterans. Obesity. 2019;27(1):161–5. 10.1002/oby.22350. 30421849 10.1002/oby.22350 PMC6309247

[26] Rippolone JE. Evaluating the Utility of Coarsened Exact Matching for Pharmacoepidemiology Using Real and Simulated Claims Data | American Journal of Epidemiology | Oxford Academic. *American Journal of Epidemiology*. 2020;189(6). Accessed June 2, 2022. https://academic.oup.com/aje/article/189/6/613/5679490 10.1093/aje/kwz268 PMC7368132 31845719

[27] Quan H, Sundararajan V, Halfon P, Fong A, Burnand B, Luthi JC, Saunders LD, Beck CA, Feasby TE, Ghali WA. Coding algorithms for defining comorbidities in ICD-9-CM and ICD-10 administrative data. Med Care. 2005;43(11):1130–9. 10.1097/01.mlr.0000182534.19832.83. 16224307 10.1097/01.mlr.0000182534.19832.83

[28] Li YP, Propert KJ, Rosenbaum PR. Balanced risk set matching. J Am Stat Assoc. 2001;96(455):870–82. 10.1198/016214501753208573.

[29] Rosenbaum PR, Rubin DB. Constructing a Comparator Group using Multivariate Matched Sampling methods that incorporate the Propensity score. Am Stat. 1985;39(1):33–8. 10.1080/00031305.1985.10479383.

